# To Predict the Length of Hospital Stay After Total Knee Arthroplasty in an Orthopedic Center in China: The Use of Machine Learning Algorithms

**DOI:** 10.3389/fsurg.2021.606038

**Published:** 2021-03-11

**Authors:** Chang Han, Jianghao Liu, Yijun Wu, Yuming Chong, Xiran Chai, Xisheng Weng

**Affiliations:** ^1^Department of Orthopaedic Surgery, Peking Union Medical College Hospital, Peking Union Medical College, Chinese Academy of Medical Sciences, Beijing, China; ^2^Eight-Year MD Program, Peking Union Medical College, Chinese Academy of Medical Sciences, Beijing, China

**Keywords:** machine learning, predictive model, cross-validation, hospital stay, total knee arthroplasty

## Abstract

**Background and Objectives:** Total knee arthroplasty (TKA) is widely performed to improve mobility and quality of life for symptomatic knee osteoarthritis patients. The accurate prediction of patients' length of hospital stay (LOS) can help clinicians for rehabilitation decision-making and bed assignment planning, which thus makes full use of medical resources.

**Methods:** Clinical characteristics were retrospectively collected from 1,298 patients who received TKA. A total of 36 variables were included to develop predictive models for LOS by multiple machine learning (ML) algorithms. The models were evaluated by the receiver operating characteristic (ROC) curve for predictive performance and decision curve analysis (DCA) for clinical values. A feature selection approach was used to identify optimal predictive factors.

**Results:** The areas under the ROC curve (AUCs) of the nine models ranged from 0.710 to 0.766. All the ML-based models performed better than models using conventional statistical methods in both ROC curves and decision curves. The random forest classifier (RFC) model with 10 variables introduced was identified as the best predictive model. The feature selection indicated the top five predictors: tourniquet time, distal femoral osteotomy thickness, osteoporosis, tibia component size, and post-operative values of Hb within 24 h.

**Conclusions:** By analyzing clinical characteristics, it is feasible to develop ML-based models for the preoperative prediction of LOS for patients who received TKA, and the RFC model performed the best.

## Introduction

Total knee arthroplasty (TKA) has been confirmed the most efficient treatment for improving outcomes of knee diseases such as end-stage osteoarthritis (1). Specifically, the increasing incidence rate of degenerative knee diseases requires an upsurge of demand for total knee replacement surgery in Chinese population (2, 3). Considering the operation cost of ~$8,000–10,000 for each patient in our center, a great economic burden on the medical care system may be expected in the next decades. Despite several parts that make up per patient's entire surgery expense, the length of hospital stay (LOS) accounts for the pivotal proportion of its increase. The accurate prediction of patients' LOS can help clinicians for decision-making and bed assignment planning, which thus makes full use of medical resources and decreases wasted hospital stays.

In recent years, fast-track arthroplasty has been conducted in some centers and showed great feasibility and safety (4, 5). Youssef F et al. reported multiple variables were associated with increased hospital LOS following primary TKA including age ≥80 years and Hispanic race and reviewed measures designed to decrease LOS (6). Nina et al. reported a mean LOS of 3.0 nights following the application of the fast-track rehabilitation protocol (7). In one meta-analysis of enhanced recovery after hip and knee arthroplasty, the mean LOS ranged from 2.0 to 8.5 days among 10 studies enrolled (8). However, to our knowledge, fast-track arthroplasty has been rarely applied in Chinese hospitals, and few studies have focused on the LOS after TKA in the Chinese population. Lo CK et al. reviewed 1,622 primary total knee replacements performed in Hong Kong and the mean length of hospital stay was 6.8 days (9). It is highly necessary to generalize the use of fast-track arthroplasty in China, and a predictive system for LOS will be the starting point.

There are no reported reliable models for predicting LOS of patients after TKA. Using machine learning (ML) algorithms, this study aims to investigate the potential indicators and develop effective predictive models for LOS in patients that received TKA.

## Materials and Methods

### Study Population

From June 2016 to June 2019, 1,298 patients who received TKA in Peking Union Medical College Hospital were retrospectively analyzed. The inclusion criteria included the following: (1) diagnosed with osteoarthritis independently by two orthopedic doctors which met surgery indication, (2) received unilateral primary TKA that was performed by our team, (3) accurate records of previous medical history, and (4) underwent bone density test within 90 days before operation. Patients who met one of the following criteria were excluded: (1) revision arthroplasty; (2) severe complications that could extremely prolong the LOS, such as dislocation, serious infection, and pulmonary embolism; (3) incomplete records; (4) transferred to other hospital after surgery; and (5) a second surgery within 30 days after the first one. The Ethics Review Board of Peking Union Medical College Hospital has approved this study, and all patients provided signed informed consent.

### Surgical Procedure

After being diagnosed with osteoarthritis in our hospital, patients who meet the surgery indication are given a booklet on perioperative information of knee surgery to make sure they are informed of their illness. Once patients choose to receive TKA, a preoperative evaluation is performed about 2 weeks before surgery, including necessary imaging examination, bone density test, serum hemoglobin (Hb), erythrocyte sedimentation rate (ESR), hematocrit (HCT), etc. For patients with Hb <9.0 g/dl, an extra supplement of iron and erythropoietin is required (10, 11), and surgery is considered and performed only when Hb ≥9.0 g/dl lasts for at least 1 week.

All patients enrolled in this study underwent standard TKA performed in the supine position by three orthopedists from Peking Union Medical College Hospital with more than 30-year experience. Tourniquet was commonly used during the operation, and the pressure was set at 60 kPa. Posterior cruciate stabilizing (PS) prosthesis was used in the operation. Physical exercise and continuous passive motion exercise device (CPM) were applied on postoperative day 1 until discharge, which were performed twice daily according to standard guidelines (12).

### Study Variables

A total of 36 variables were used for modeling analysis in this study. The patient-related characteristics included sex, age, body mass index (BMI), smoking status, alcohol abuse history, past surgical history, inpatient surgery history, and comorbidities. Comorbidity burden was considered based on the Clinical Modification of the ninth version of the International Classification of Diseases (ICD-9-CM) codes and the comorbidity distribution in our studied population. It was made up of several categories, each corresponding to a group of specific diagnoses, including the following: heart diseases, pulmonary diseases, liver diseases, renal diseases, peripheral vascular diseases, digestive diseases (excluding bleeding), cerebrovascular diseases, neoplastic diseases, ophthalmological disease, connective tissue diseases, hypertension, diabetes mellitus, hyperlipidemia, and osteoporosis. All the above mentioned clinical information was collected before surgery. The diagnosis of osteoporosis was confirmed by senior orthopedists reviewing the result of the bone density test.

The perioperative factors we analyzed included serum Hb, HCT, ESR, anesthesia method (general anesthesia, general anesthesia plus nerve block and others), intraoperative blood loss (IBL), actual blood loss (ABL), blood transfusion volume (BTV), tourniquet time (TQT), postoperative drainage volume (PDV), femoral component size (FCS), tibia component size (TCS), insert thickness (IT), distal femoral osteotomy thickness (FOT), and tibial plateau osteotomy thickness (TOT). Preoperative Hb (pre-Hb), HCT (pre-HCT), and ESR (pre-ESR) were tested within 24 h before surgery, while postoperative values of Hb were within 24 h (post-Hb-1) and 48 h (post-Hb-2) after surgery, respectively. The prostheses used were mainly provided by two different manufacturers. According to the size of the prostheses, patients were divided into a small group (<25%), normal group, and large group (>75%). All patients were hospitalized longer than 4 days, so some intraoperative and early postoperative indicators such as post-Hb-1 were included.

ABL was estimated with the Gross equation and, thus, could be acquired by adding the autologous blood transfusion and the allogenetic blood transfusion. The specific calculation method for the Gross equation is as follows: 2^*^PBV × (pre-operative HCT – post-operative HCT)/(pre-operative HCT + post-operative HCT). Finally, patients were divided into two groups according to the median days of LOS.

### Development and Validation of ML Models

The ML algorithm is a very useful tool on data mining and has been widely applied in medicine (13, 14). In the process of supervised ML, the algorithm learns from the labeled data. After understanding the data, it will determine which label should be assigned to the new data based on the pattern, and then associate the pattern with the unlabeled new data. In this study, seven typical supervised ML algorithms were used to develop predictive models for LOS in patients who received TKA, namely adaptive boosting (ADB), artificial neural network (ANN), K-nearest neighbor (KNN), decision tree (DT), gradient boosting decision tree (GBDT), random forest classifier (RFC), and extreme gradient boosting (XGB) (15–21). In addition, two conventional methods, logistic regression (LR) and multinomial naïve Bayes (MNB), were used for comparing with ML models. Many machine learning algorithms perform best on normally distributed datasets, and LOS was following a non-normal distribution and log-transformed before analyses.

Before constructing the models, data preprocessing is necessary for continuous variables. Therefore, min–max normalization was used for MNB, while *z*-score normalization was used for others (22). The highest number of missing values was in the variable “tourniquet time,” which had missing values in 3.2% of the items. The missing data was within the acceptable range and solved by imputing median values (23, 24).

Considering a large number of variables introduced, the 5-fold cross-validation strategy was used to avoid overfitting. All patients were randomly split into five subsets, one of which worked as the validation cohort and the other four as the training cohort for each iteration. This process was repeated five times, and so, each subset acted once as a validation cohort. In the end, the optimal model of each algorithm was returned. To evaluate the models, the area under the receiver operating characteristic (ROC) curve (AUC) was used to compare predictive ability, while decision curve analysis (DCA) was performed to investigate clinical usefulness.

### Feature Selection

Given the invisible function between the variables and the response in most of the ML algorithms in our study, a classifier evaluator was used to measure each variable's predictive contribution for feature selection. For each constructed model, a list of variables with ranks was returned. The higher the rank, the greater the predictive contribution to the model. The top 10 variables among the excellent models were shown. In addition, the AUCs of each model with a different number of variables introduced were also calculated to help select the optimal dimension.

### Statistical Analysis

All statistical analyses and ML model development were performed using Python programming language (version 3.7, Python Software Foundation). The continuous variables were expressed as median with interquartile range (IQR) because of their non-normality tested by the Shapiro–Wilk method, while the classified variables were expressed as frequency with percentage. The comparisons of continuous variables between subgroups were conducted by the Mann–Whitney *U*-test, and the classified subgroups were compared by chi-square test (Fisher's exact test was used when necessary). Student's *t*-test was applied for the comparisons of AUCs between different models. A two-sided *P*-value <0.05 was considered significant for all analyses.

## Results

### Demographic Characteristics

All 1,298 patients' clinical characteristics are listed in Table 1. The mean of LOS was 8.4 days. In total, the mean BMI was 26.8 and 1,022 (78.7%) patients were male. One hundred and six (8.2%) patients reported smoking cigarettes in the year before admission for TKA. Fifty-six (4.3%) patients had an alcohol abuse problem. The most common comorbidities identified were osteoporosis (429, 33.1%), hypertension (699, 53.9%), and diabetes mellitus (244, 18.8%). Twenty-four (1.8%) patients received blood transfusion.

**Table 1:** Patients' clinical characteristics.

**Characteristics**	**Mean/count (SD/%)**
Age, years	67.6 (9.3)
Sex	
Male	1,022 (78.7)
Female	276 (21.3)
Body mass index (BMI)	26.8 (3.8)
Smoking history	106 (8.2)
Alcohol abuse history	56 (4.3)
Comorbidities	
Osteoporosis	429 (33.1)
Hypertension	699 (53.9)
Hyperlipidemia	123 (9.5)
Diabetes mellitus	244 (18.8)
Hematological diseases	27 (2.1)
Ophthalmological disease	57 (4.4)
Renal disease	55 (4.2)
Heart disease	188 (14.5)
Digestive disease	89 (6.9)
Connective tissue disease	74 (5.7)
Pulmonary disease	68 (5.2)
Neoplastic disease	58 (4.5)
Cerebrovascular disease	100 (7.7)
Liver disease	43 (3.3)
Preoperative Hb, g/dl	13.2 (12.4)
Preoperative HCT, %	39.1 (3.3)
Preoperative ESR, mm/h	15.8 (12.7)
Blood transfusion	24 (1.8)
Tourniquet time, min	82.1 (11.7)
Insert thickness, mm	9.7 (2.6)
Distal femoral osteotomy thickness, mm	9.8 (1.2)
Tibial plateau osteotomy thickness, mm	8.7 (1.2)
Length of hospital stay, days	8.4 (4.6)

### Predictive Performance of ML Models

**Figure 1:**
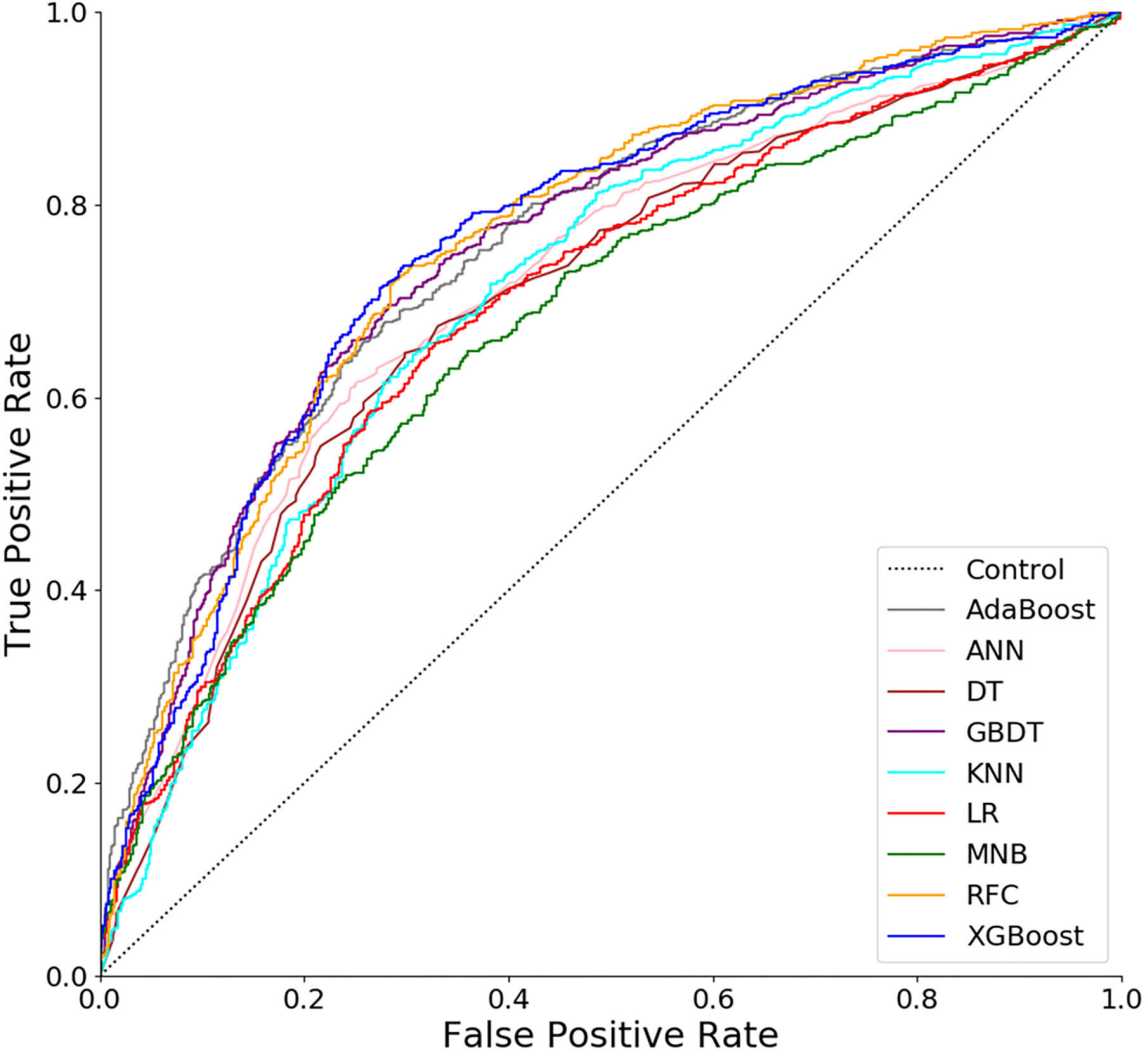
Receiver operating characteristic (ROC) curves for nine models. ADB, adaptive boosting; ANN, artificial neural network; KNN, K-nearest neighbor; DT, decision tree; GBDT, gradient boosting decision tree; RFC, random forest classifier; XGB, extreme gradient boosting; LR, logistic regression; MNB, multinomial naïve Bayes.

As shown in Figure 1 and Table 2, the RFC model performed best in the ROC curve (AUC = 0.766, 95% CI: 0.714–0.817), which presented a similar AUC value to XGB (AUC = 0.762, 95% CI: 0.710–0.814, *P* = 0.850), GBDT (AUC = 0.758, 95% CI: 0.705–0.810, *P* = 0.668), and ADB (AUC = 0.757, 95% CI: 0.704–0.809, *P* = 0.647), but was superior to ANN (AUC = 0.717, 95% CI: 0.662–0.772, *P* = 0.012), DT (AUC = 0.713, 95% CI: 0.657–0.768, *P* = 0.006), and KNN (AUC = 0.710, 95% CI: 0.655–0.766, *P* = 0.004). All ML models performed better than the two models using conventional LR (AUC = 0.701, 95% CI: 0.645–0.757, *P* < 0.001) and MNB (AUC = 0.684, 95% CI: 0.627–0.741, *P* < 0.001).

**Table 2:** Predictive performance (AUC) of 8 models and using several variables alone.

**Model**	**AUC**				**No. of optimal dimensions**
	**Mean**	**SD**	**95% CI**	***P*-value**	
ADB	0.757	0.026	0.704–0.809	0.647	21
ANN	0.717	0.028	0.662–0.772	0.012	7
DT	0.713	0.028	0.657–0.768	0.006	5
GBDT	0.758	0.026	0.705–0.810	0.668	18
kNN	0.71	0.028	0.655–0.766	0.004	19
LR	0.701	0.028	0.645–0.757	<0.001	28
MNB	0.684	0.029	0.627–0.741	<0.001	19
RFC	0.766	0.026	0.714–0.817	–	18
XGB	0.762	0.026	0.710–0.814	0.850	19

### Clinical Usefulness of ML Models

**Figure 2:**
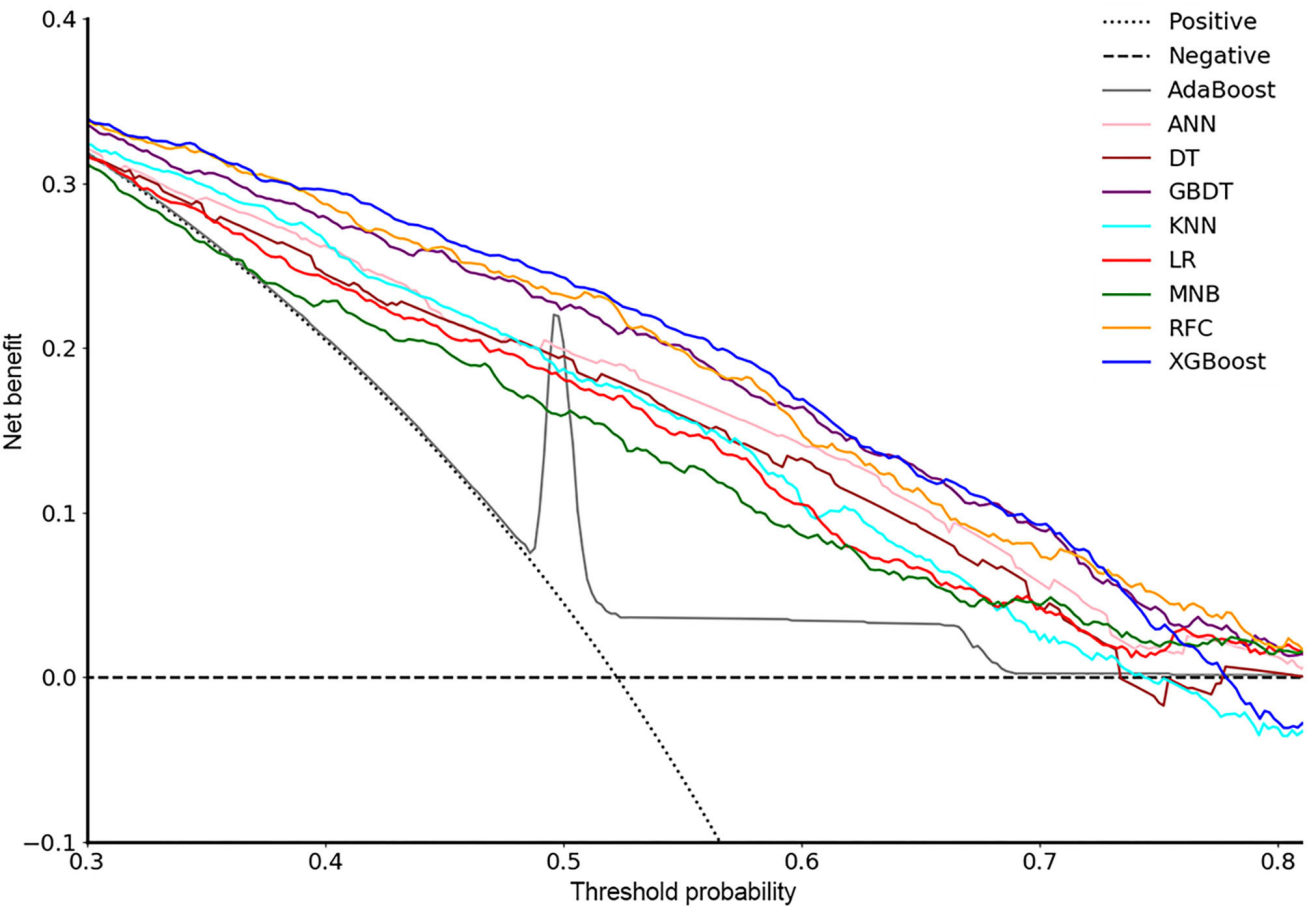
Decision curve for nine models. ADB, adaptive boosting; ANN, artificial neural network; KNN, K-nearest neighbor; DT, decision tree; GBDT, gradient boosting decision tree; RFC, random forest classifier; XGB, extreme gradient boosting; LR, logistic regression; MNB, multinomial naïve Bayes.

In the decision curve (Figure 2), all models we developed performed much better than the two extreme lines except ADB, which overlapped with the extreme lines in most of the thresholds. Almost under all reasonable circumstances, XGB, RFC, and GBDT showed higher net benefits than the other models. The clinical usefulness of conventional LR and MNB was also inferior to most of the ML models.

### Variable Importance

**Figure 3:**
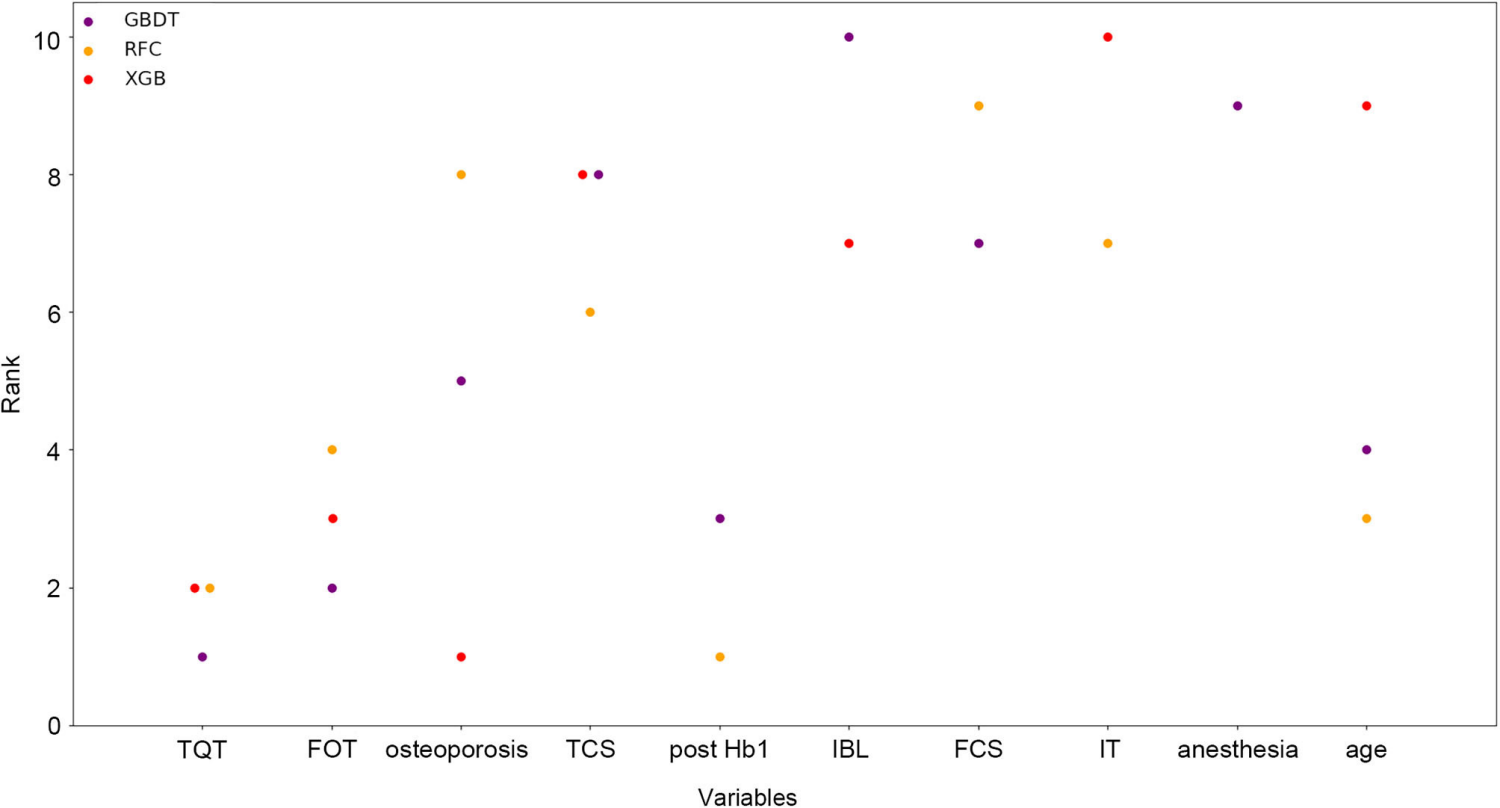
Ranks of the top 10 variables for the prediction of length of hospital stay (LOS). Variables were ranked using a classifier-specific evaluator based on machine learning algorithms. Each variable was ordered according to their mean ranks. The lower rank represents more contributions to the prediction of LOS. For example, tourniquet time was ranked 1st, 2nd, and 3rd in GBDT, RFC, and XGB, respectively. TQT, tourniquet time; FRT, femoral resection thickness; TCS, tibia component size; IBL, intraoperative blood loss; FCS, femoral component size; IT, insert thickness.

For each model, all variables were ranked based on their contributions to the predictive performance. In Figure 3, the 10 top-ranked variables were shown according to their mean ranks among the most potential models (RFC, XGB, and GBDT), which demonstrated better performance in both ROC and decision curves than the other models. The 10 variables were as follows: tourniquet time, distal femoral osteotomy thickness, osteoporosis, tibia component size, post-Hb-1, intraoperative blood loss, femoral component size, insert thickness, anesthesia, and age.

### Dynamic Application Based on RFC

**Figure 4:**
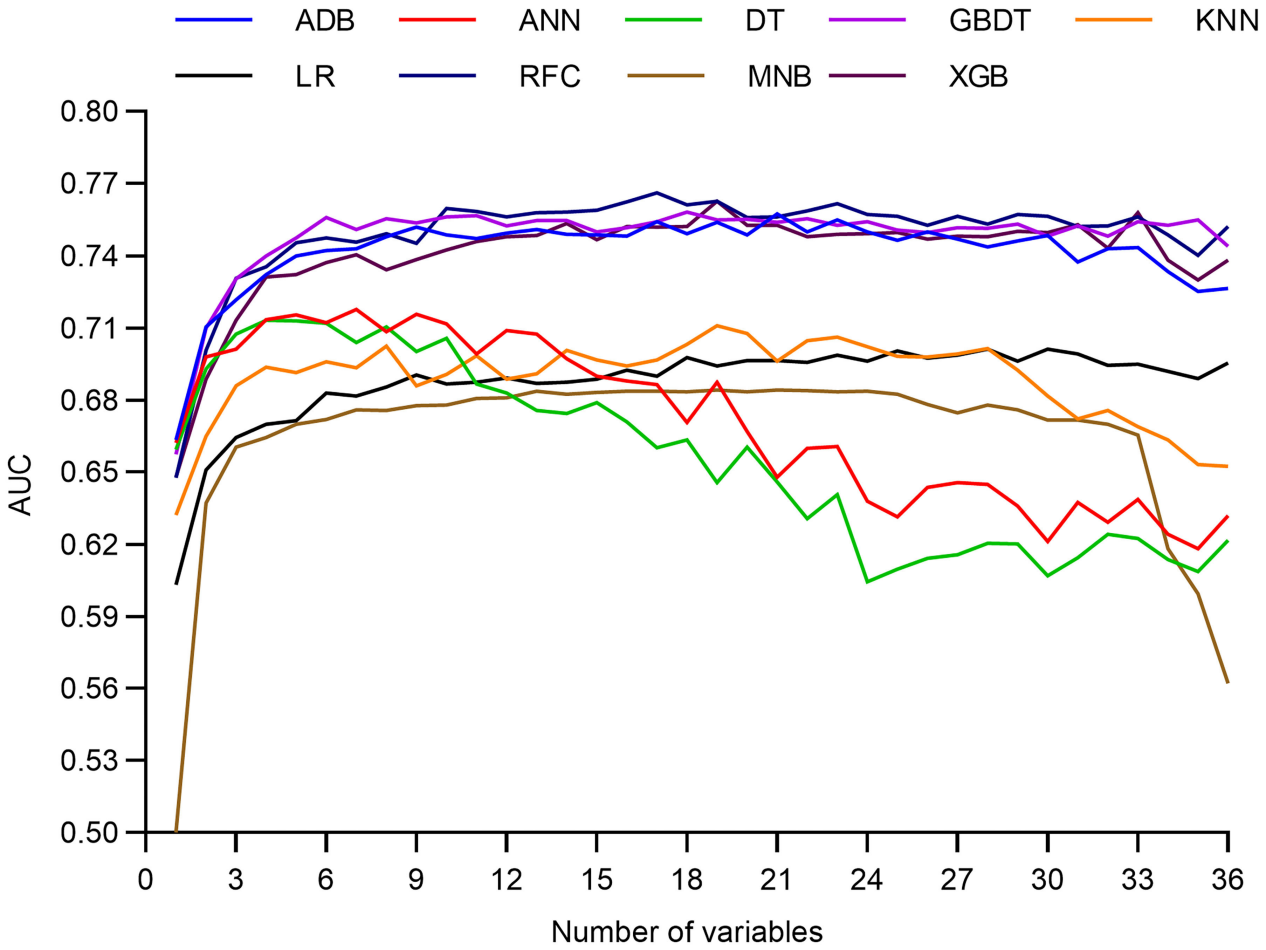
Predictive performance of the random forest classifier (RFC) model at each number of variables. ADB, adaptive boosting; ANN, artificial neural network; KNN, K-nearest neighbor; DT, decision tree; GBDT, gradient boosting decision tree; RFC, random forest classifier; XGB, extreme gradient boosting; LR, logistic regression; MNB, multinomial naïve Bayes.

**Figure 5:**
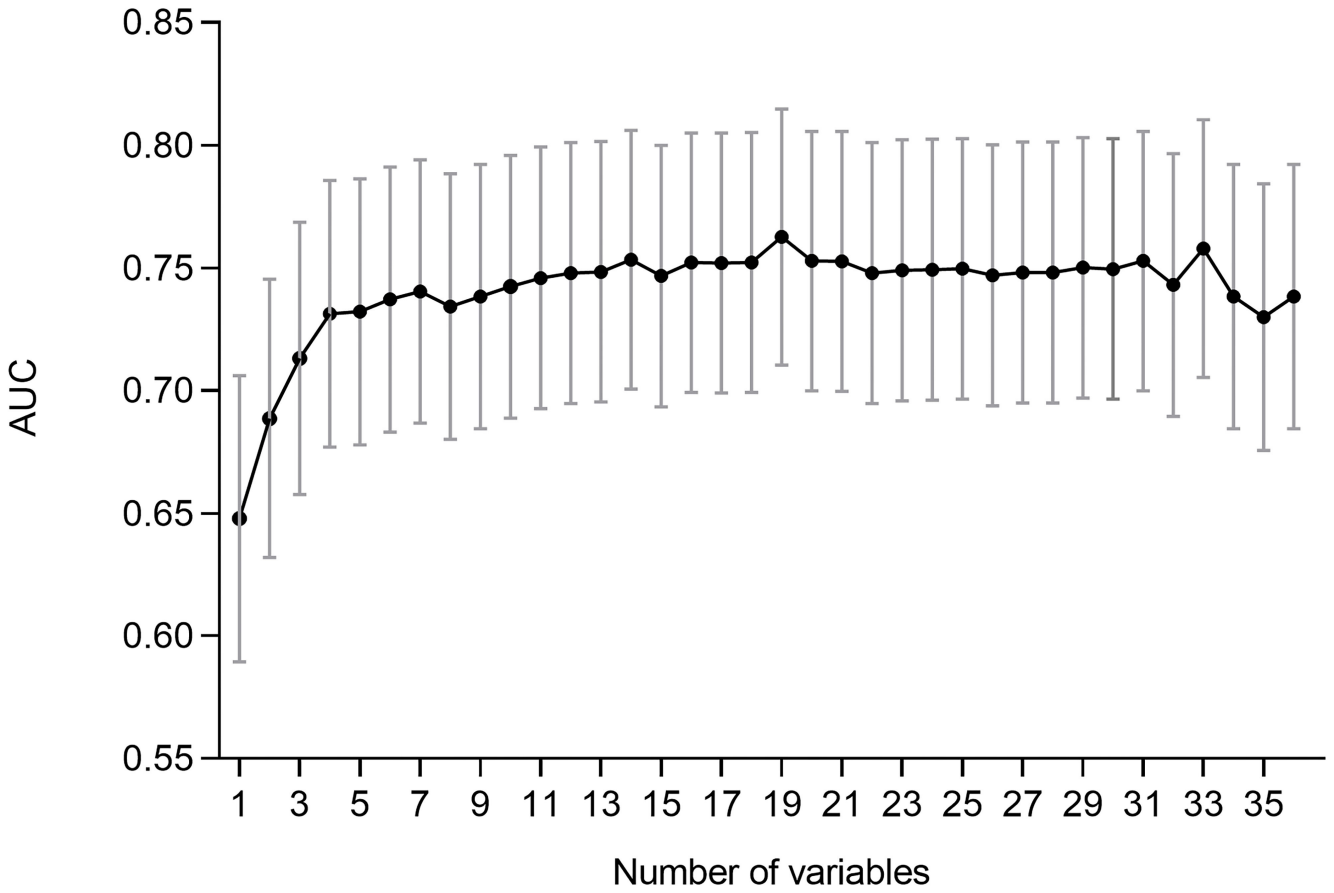
Predictive performance (AUCs) of the random forest classifier (RFC) model at each number of variables.

**Figure 6:**
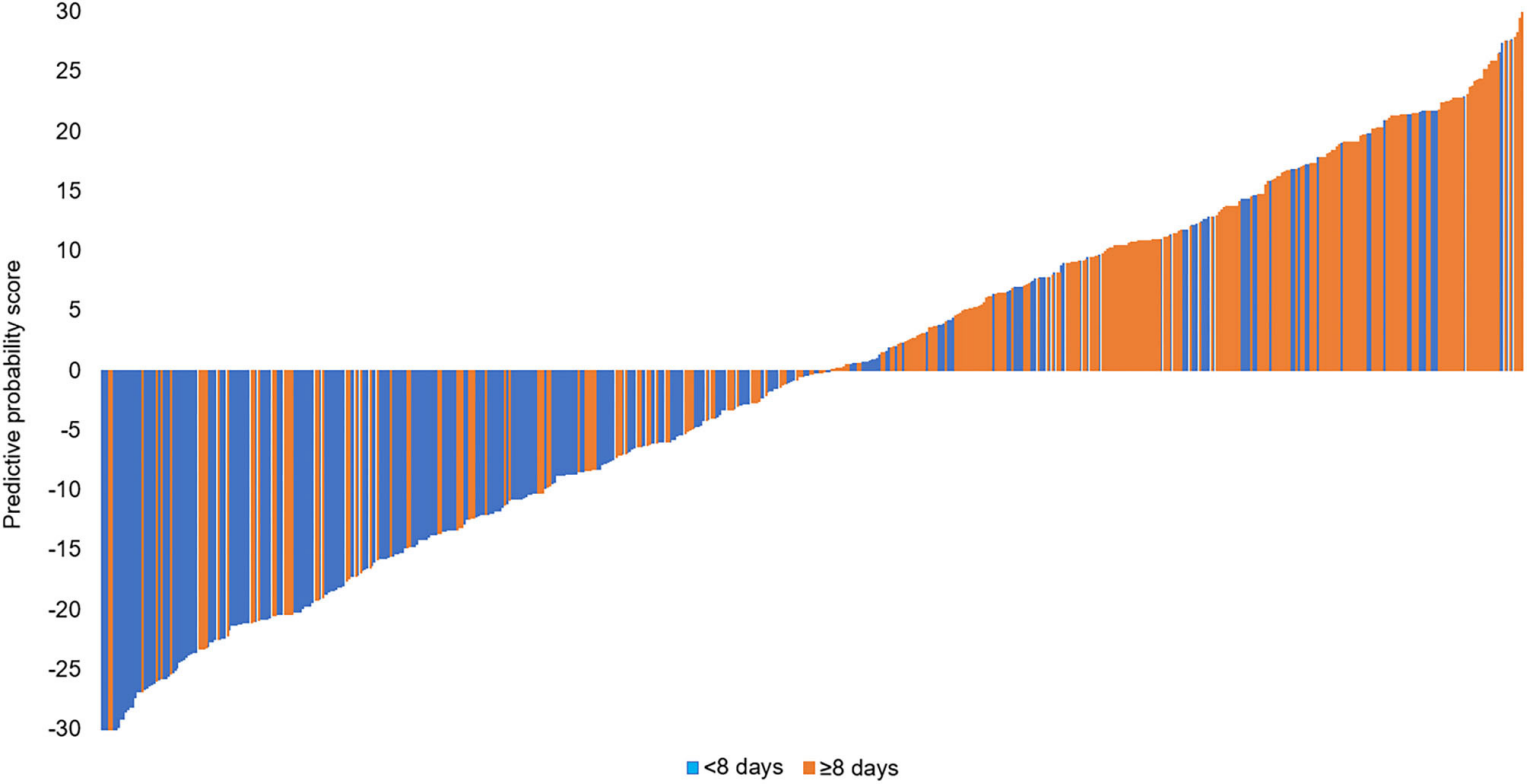
Three hundred patients were randomly drawn from each group of LOS <8 days and LOS ≥8 days. Predictive probability calculated for 300 patients (150 <8 days, 150 ≥8 days).

Since the RFC model achieved highest AUC value with fewer number of variables introduced than XGB and GBDT (Figure 4 and Table 2), the RFC algorithm was considered for developing a dynamic application for predicting the LOS of each patient in the clinic. It can be noted that the RFC model's AUC value reached the plateau with 10 variables introduced and did not change a lot from the 10 (AUC = 0.760) to the 17 variables (AUC = 0.766; Figure 5). Thus, a dynamic application based on the RFC model with the 10 variables introduced was developed. The optimal cutoff point to distinguish LOS <8 days and LOS ≥8 days was 0.522 according to the maximal Youden's index in the RFC model. Three hundred patients were randomly drawn from each group of LOS <8 days and LOS ≥8 days. Using the RFC model application, a patient's risk probability plus 95% CI of LOS ≥8 days can be obtained immediately when imputing information of the 10 variables we identified, and each patient's predictive probability was standardized by the following formula: (probability – 0.522)/standard deviation (Figure 6). The *x*-axis represents each patient, while the *y*-axis represents the standardized probability score from the RFC model.

## Discussion

TKA is widely performed to improve mobility and quality of life for symptomatic knee osteoarthritis patients. More than 500,000 knee replacements are performed annually in the USA (25). This number is projected to grow by 673% from 2005 to 2030 (26). A greater number of frail older people with complex comorbid conditions now receive knee arthroplasty. Implementation of fast-track arthroplasty resulted in a shorter length of hospital stay and reductions in complications, while readmissions and costs are reduced (27).

In our study, incorporating a total of 36 variables from 1,298 patients, we firstly developed predictive models for LOS after TKA in a Chinese population. Seven representative supervised ML algorithms and two conventional methods were applied. In the ROC curve (Figure 1), all ML-based models showed much higher AUC values than two models using conventional methods (LR and MNB), and four of them (RFC, XGB, GBDT, and ADB) performed significantly better than the others. Also, in the decision curve (Figure 2), most of the ML models presented much more clinical values and could make patients benefit more. Thus, from the two aspects of predictive ability and clinical usefulness, ML algorithms performed better than the conventional methods. According to this study, RFC, XGB, and GBDT were identified as the most excellent and potential algorithms for model development because of their better performance in both ROC and decision curves.

Prediction performance is crucial to predictive models, indicating the ability to distinguish between positives and negatives, which can be evaluated and compared by the AUC values. However, the models with high AUC might not be clinically useful (for example, the ADB model in our study). Thus, it is also important to assess the models' clinical utility by DCA, which can consider the net benefits of all patients (28). All ML models presented much higher net benefits than the treat-none and treat-all lines. This means that, according to the predictive outcome of the ML models, clinicians could take necessary interventions for those who were predicted to have longer LOS, such as more observations, intensive care, more active medical strategies, and reasonable bed assignment plans, to maximize the net benefits of all patients. Obviously, the three models we identified, RFC, XGB, and GBDT, were more useful for clinical decision-making and more beneficial to the patients (Figure 2).

Previous studies have found several factors that might be associated with longer LOS by conventional statistical analysis. In our study, feature selection strategy was used. Based on the mean ranks of each variable among the three most potential models, we identified the top 10 variables that contributed most to predictive performance (Figure 3).

Patients with longer tourniquet time showed longer LOS in our study. A systematic review found that tourniquet use reduced intraoperative blood loss (198 ml) and operating time (5 min) (29). However, Mizner et al. (30) found that quadriceps strength was 62% less than pre-operative values when measured 4 weeks after TKA. Liu et al. (31) found that tourniquet patients had significantly less quadriceps muscle activity on EMG for the first 6 months postoperatively as well as increased pain on days 2 and 4 post-operatively compared with non-tourniquet patients. This result may be explained by the fact that the ischemic conditions brought about by the use of a tourniquet could induce sustained local reactive hyperemia lasting several hours after the tourniquet deflation (32, 33). Another possible alternative explanation is that the increased fibrinolytic activity associated with tourniquet-induced ischemia promotes bleeding into the local tissues following the procedure (34, 35). Patients who are still in pain and are not functioning well after surgery are usually kept in the hospital until they are fit enough to be sent home.

Today, orthopedic surgeons could offer TKAs to older and more complex patients (36, 37). This is mainly attributed to the recent advances in medical management and the faster rehabilitation processes. Some older patients are more medically complex requiring longer hospital stays for medical optimization prior to discharge. This is manifested in this study as age was associated with a prolonged LOS and it was in accordance with other studies (4, 38, 39) on LOS after TKA.

ASA provides a classification system for the fitness of a patient; however, it does not take into account all relevant comorbidities possibly associated with a prolonged LOS. A previous study about comorbidities with longer LOS included neurological comorbidities (40), preoperative anemia (41), cardiac disease, and pulmonary disease (4). In our study, based on our experience at the community hospital and expert opinion, we included 16 kinds of relevant comorbidities. An important finding of the present study was that an increased LOS was associated with osteoporosis. One half of US women and a fifth of men over 50 years suffer an osteoporotic fracture during their lifetime (42). As the population ages, more patients with osteoporosis will require orthopedic procedures, including arthroplasty. A range of adverse outcomes are more likely to arise in the osteoporotic patient after TKA including intraoperative fracture, periprosthetic osteolysis with implant migration, and postoperative periprosthetic fracture (43). This finding may help us to prioritize the optimization of bone strength and quality in perioperative.

Patients who use bigger tibia component, femoral component, or thicker distal femoral osteotomy thickness were likely to stay longer in hospital. Nagai K et al. (44) found that postoperative active flexion angle was negatively correlated with the osteotomized bone thickness of femoral medial posterior condyle (*R* = −0.37, *P* = 0.012); it means osteotomized bone thickness significantly affected the postoperative active knee flexion in the early term after PS TKA. Yue B et al. (45) found that Chinese females have a significantly narrower distal femur than white females. Furthermore, the osteoarthritic knees requiring TKA, which have been measured by many studies, are deformed according to different disease stages and frequently have anatomical dimensions different from normal knees (46, 47). These studies suggested that a greater range of femoral implant sizes may be necessary to accommodate femoral aspect ratio variations specific to Asian knees.

According to the median LOS of 8 days, this study divided the LOS into two groups: <8 and ≥8 days. Short hospital stay reduces the risk of intrahospital infection and reduces the costs. In China, there is a shortage of medical resources. Actually, Beijing and Shanghai with the most advanced medical resources and techniques have attracted a large proportion of patients living in other regions of China. Our care system is struggling to improve the quality of health care while treating more patients. This LOS model can better help patients understand hospital stay duration and help doctors make care decisions and reasonable bed assignment plans. This also revealed that the promotion of fast-track arthroplasty in China would be very meaningful.

Based on the best model (RFC with 10 variables introduced), we developed a dynamic application, which is available to all clinicians and patients. Using the application, the LOS of each patient can be precisely predicted, returning the probability of LOS ≥8 days. Thus, the application and our study would be the starting point of popularizing fast-track arthroplasty in Chinese hospitals, which is expected to save a large amount of medical expenses in the future.

Besides clinical indications, several methodological innovations should also be noted in this study. ML algorithms have been proven to be superior to conventional statistics on data mining, which was also consolidated by our study. To our knowledge, very few previous studies have developed ML-based models for predicting LOS. Our study demonstrated the feasibility of using ML algorithms. In the future, this novel tool can be used for developing more predictive models for clinical application.

There were also several limitation points that should be noted. First, LOS is affected by psychological, economic, social, and multiple medical factors of patients. It is difficult to draw a clear causal relationship. Second, this study is based on retrospective data; thus, data or systematic bias could not be totally avoided. Third, we only enrolled patients in our hospital. To develop more reliable predictive models that can be applied in Chinese hospitals, multicenter cohorts are required in the future.

## Conclusions

Using ML algorithms, it is feasible to predict the LOS of patients who received TKA. The RFC with 10 variables introduced was considered to be the optimal model and an application based on it was developed to help in clinical decision-making, which may promote the application of fast-track arthroplasty in Chinese hospitals.

## Data Availability Statement

The raw data supporting the conclusions of this article will be made available by the authors, without undue reservation.

## Ethics Statement

The studies involving human participants were reviewed and approved by Peking Union Medical College Hospital Ethics Committee. The patients/participants provided their written informed consent to participate in this study.

## Author Contributions

CH and JL: conception and design. CH and YW: provision of study materials or patients. CH, YC, and XC: collection and assembly of data. CH and XC: data analysis and interpretation. All authors: manuscript writing and final approval of the manuscript.

## Conflict of Interest

The authors declare that the research was conducted in the absence of any commercial or financial relationships that could be construed as a potential conflict of interest.
